# Targeting cancer cell mitochondria with a multivalent source of singlet oxygen

**DOI:** 10.1002/smo2.70035

**Published:** 2026-02-10

**Authors:** Wanwan Wang, Lei Wang, Rensong Sun, Shoucai Yan, Engin U. Akkaya

**Affiliations:** ^1^ Department of Pharmaceutical Engineering State Key Laboratory of Fine Chemicals Dalian University of Technology Dalian China; ^2^ Department of Chemistry Bilkent University Ankara Turkey

**Keywords:** cancer, endoperoxides, mitochondria targeting, PDT, singlet oxygen

## Abstract

Naphthalene, anthracene and pyridone endoperoxides are known to thermally release singlet oxygen. However, in the cycloreversion reaction, singlet oxygen is produced stoichiometrically; therefore, multiple singlet oxygen releasing modules are expected to be very useful in inducing apoptosis of cancer cells. Herein, we present a potential therapeutic agent presenting three‐pyridone endoperoxide modules and a mitochondria targeting group. Compared to previously reported pyridone‐based monofunctional endoperoxides, the triple endoperoxide is highly effective as evidenced by assays and fluorescence microscopy.

## Graphical abstract

Multivalent source of singlet oxygen can effectively target cancer cell mitochondria and cause improved cancer cell selective cytotoxicity at low micromolar concentrations.

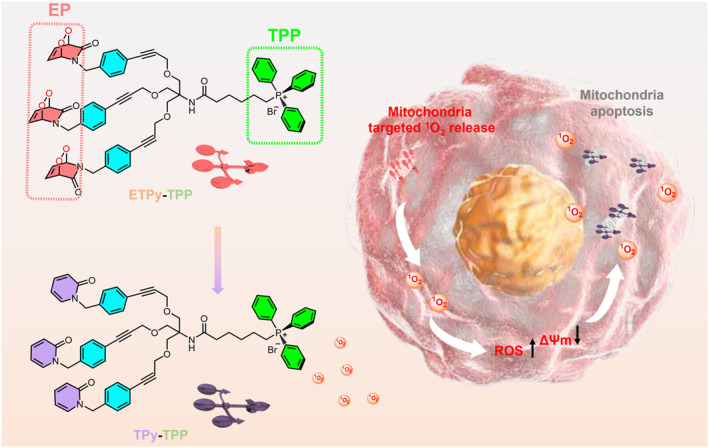

## 1 INTRODUCTION

Photodynamic therapy (PDT), while proving itself as a useful tool in treating certain superficial tumors accessible by external light sources, is very limited otherwise by the absorption and scattering of light in tissues in the relevant regions of the electromagnetic spectrum.[^1^, ^2^] Highly impeded transmission of light has limited the progress in the field for many decades. As a potential solution, our research group has been exploring[^3^, ^4^, ^5^, ^6^, ^7^, ^8^, ^9^, ^10^] the utility of chemically generated singlet oxygen in the vicinity or inside the tumors, to initiate essentially the same biological response to PDT, but without any dependence on light (and oxygen, for that matter). Most appropriate sources of singlet oxygen for this purpose are cycloaddition products of singlet oxygen to various aromatics[^11^, ^12^] and 2‐pyridone derivatives.[^13^, ^14^] These endoperoxides undergo thermal cycloreversion reactions, regenerating the precursor aromatic compound and singlet oxygen. Our results using these metastable sources of singlet oxygen are highly promising, but there are still issues to be explored, regarding the rate and the amount of the singlet oxygen to be released. In this work, our aim was to improve the effectiveness mitochondria targeted pyridone endoperoxides and efficacy of the singlet oxygen donation by proving multiple endoperoxide modules in a unimolecular construct.

## 2 RESULTS AND DISCUSSION

### 2.1 Design of singlet oxygen delivery agent

**FIGURE 1:**
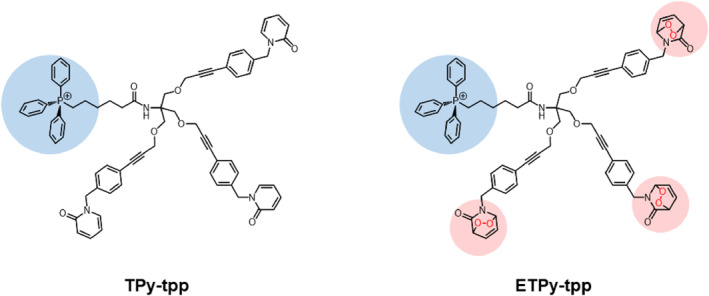
Structures of control pyridone compound (**TPy‐TPP**) and the endoperoxide (**ETPy‐TPP**). TPP groups conferring mitochondria targeting properties^[15]^ were identified in blue circles, and the singlet oxygen releasing pyridone endoperoxide modules are highlighted in pink circles.

**Scheme 1:**
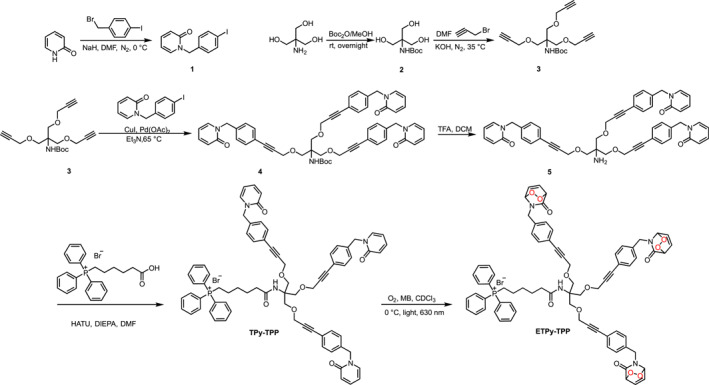
The synthesis of **ETPy‐TPP**.

In our recent work, we demonstrated that the delivery of singlet oxygen to cancer cell mitochondria is very effective, reducing IC_50_ up to two orders of magnitude. With this consideration, we incorporated triphenylphosphonium (TPP) moiety in the target compound **ETPy‐TPP**) (Figure 1). Ethynyl linkers, which facilitate synthesis, are known to be unaffected by singlet oxygen.^[16]^ The branching linker we used in the design is the common buffer compound TRIS (trishydroxymethyl)methylamine. The detailed synthesis procedures are available in the experimental section (Scheme 1).

### 2.2 Cycloreversion reaction

The cycloreversion of the endoperoxide **ETPy‐TPP** to the precursor 2‐pyridone derivative **TPy‐TPP** was studied by ^1^H NMR at 37°C in CDCl_3_ (Figures S1 and S2). The half‐life was calculated to be 4.5 h. All three pyridone endoperoxide modules behave essentially identical in terms of reaction rate, as the modules are distant from each other and there is no steric or electronic interaction between them. Singlet oxygen release was confirmed (Figure S3) using the selective probe diphenylisobenzofuran (DPBF) (Figures S4–S6).

### 2.3 MTT and live‐dead assays; fluorescence imaging of singlet oxygen

**FIGURE 2:**
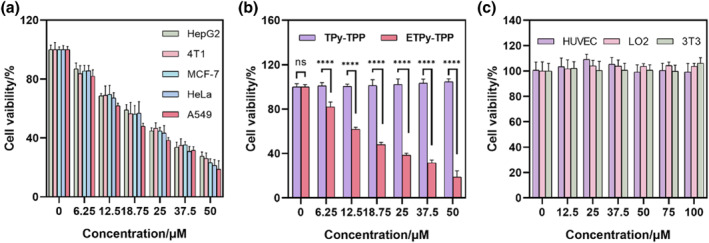
MTT assay: (a) Cell viability of multiple cancer cell lines treated with **ETPy‐TPP** at various concentrations; (b) Cell viability of A549 cells treated with **TPy‐TPP** and **ETPy‐TPP** at various concentrations; (c) Cell viability of multiple normal cell lines treated with **ETPy‐TPP** at various concentrations.

The cytotoxic activity of the endoperoxide **ETPy‐TPP** and the control compound **TPy‐TPP** were studied using MTT assays A549, MCF‐7, HepG2, HeLa, 4T1 cell lines (Figure 2a). For **ETPy‐tpp** IC_50_ values are approximately 20 μM for all cell lines studied (18.3, 22.4, 23.2, 21.3, 22.3 μM for A549, MCF‐7, HepG2, Hela, 4T1, respectively). This number is much smaller, indicating more effective delivery of singlet oxygen compared to comparable monovalent sources of singlet oxygen.^[17]^ **TPy‐TPP** has no cytotoxicity at the concentrations studied (Figure 2b). Even more impressive is the fact that the endoperoxide compound does not display cytotoxicity towards normal cells (Figure 2c).

**FIGURE 3:**
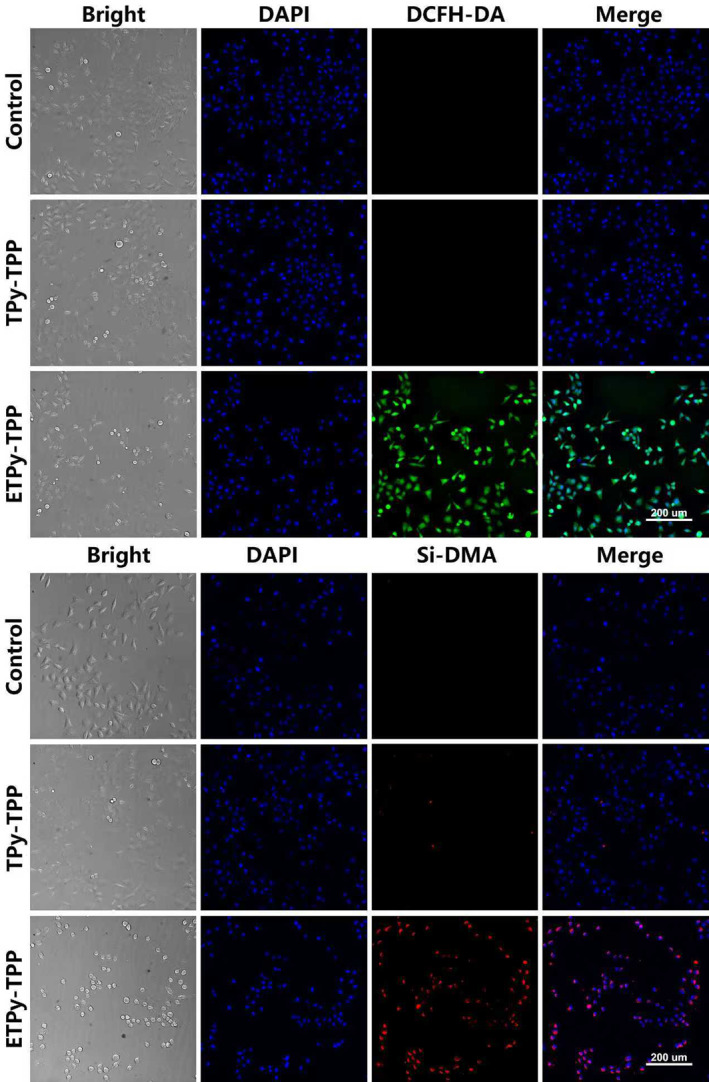
Intracellular singlet oxygen generation with 30 μM agents in A549 cells as probed by DCFH‐DA (top) and Si‐DMA (bottom).

Intracellular singlet oxygen release was imaged under fluorescence microscopy using reactive oxygen species (ROS) probe DCFH‐DA (Figure 3), the results show that only **ETPy‐TPP** acts as a source of singlet oxygen, as expected has no cytotoxicity at the concentrations studied. We also checked for more specific release of singlet oxygen inside the mitochondria using mitochondrial singlet oxygen probe Si‐DMA. It is clear that the endoperoxide compound releases singlet oxygen in cancer cell (A549) mitochondria. Similarly, mitochondrial membrane potential was examined using the dye JC‐1. The aggregate structure of the dye has longer wavelength emission (orange‐red) but as the membrane potential is diminished by the action of singlet oxygen released in mitochondria, green monomer emission becomes predominant. This is observed when the cells are exposed to **ETPy‐TPP** (Figure S7).

The live/dead staining assay was performed to visualize the viability of A549 cells after being treated with different chemical agents. As expected, exposure to **ETPy‐TPP** kills the cells, whereas **TPy‐TPP** has no discernable effect (Figure S8).

In addition, tumor spheroids were prepared using A549 cells for live‐dead assays in 3D cell cultures. The results clearly confirm the strong anti‐tumor action of the endoperoxide compound (Figure S9).

### 2.4 Colony formation assays, flow cytometry, invasiveness assays

**FIGURE 4:**
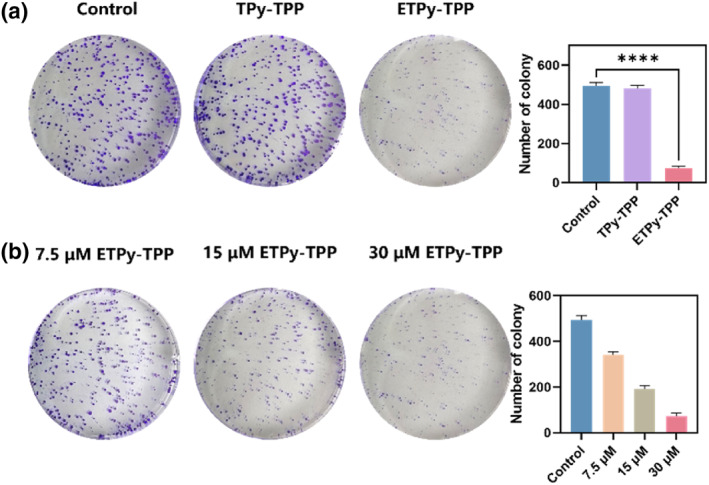
(a) The effects of **TPy‐TPP** and **ETPy‐TPP** on the colony formation assay of A549 cells and the number of cloned cell colonies. (b) The effect of different concentrations of **ETPy‐TPP** on cell cloning and the number of cloned cell colonies.

Colony formation assays, in many cases, are considered to be a more robust indicator of the drug efficacy. **ETpy‐TPP** decreased the colony number down to less than half of the control at 15 μM (Figure 4).

**FIGURE 5:**
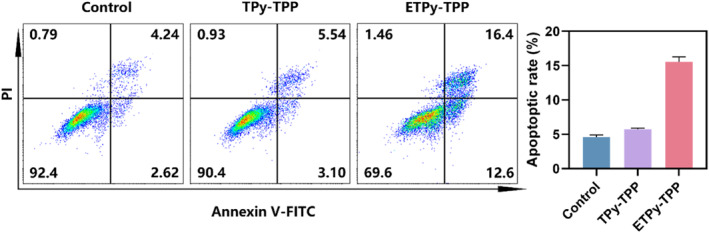
Flow cytometry analysis of apoptosis after treatments with 30 μM **TPy‐TPP** and **ETPy‐TPP**.

Flow cytometry analysis (Figure 5) shows that Q2 region is significantly enhanced compared to the control, indicating apoptotic transformation with **ETpy‐TPP**. **Tpy‐TPP** on the other hand has negligible effect.

**FIGURE 6:**
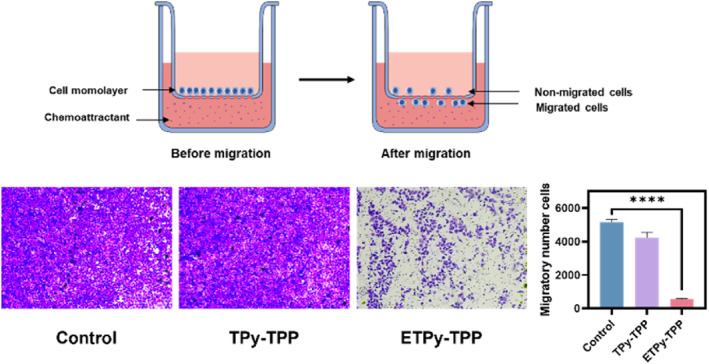
Transwell migration assay of A549 cells and schematic representation of the principle, representative images of cell migration after treatments with 30 μM **TPy‐TPP** and **ETPy‐TPP**, and quantitative analysis of migratory capacity. Scale, 100 μm.

Scratch tests (Figure S10) and transwell migration tests (Figure 6) showed that cell migration and invasiveness of the cancer cells significantly diminished on exposure to the endoperoxide compound. This is also in line with our expectations.

## 3 EXPERIMENTAL SECTION

### 3.1 Materials

Unless otherwise specified, all reagents and solvents were purchased from commercial suppliers and used without further purification. Reactions were monitored by thin layer chromatography using Huang‐hai TLC Silica gel 60 F‐254. Column chromatography was performed by using Mei‐gao Silica Gel 60 (particle size: 200–300 mesh). 1,3‐Diphenylisobenzofuran (DPBF) was purchased from Shanghai aladdin Co., Ltd. Reactive oxygen species Assay Kit, Mitochondria Membrane Potential Assay Kit and Calcein‐AM/PI Live/Dead Cell Double Stain Kit was bought from Beijing Solarbio Science & Technology Co., Ltd. Si‐DMA for Mitochondrial Singlet Oxygen Imaging was bought from Dojindo Molecular Technologies, Inc. DAPI staining solution and Annexin V‐FITC Apoptosis Detection Kit were bought from Shanghai Beyotime Biotechnology Co., Ltd (Shanghai, China).

### 3.2 Instruments

The ^1^H and ^13^C NMR spectra were recorded using Bruker Vaian DLG400 (Figure S11). Chemical shifts were reported in parts per million (ppm) and coupling constants (J values) are given in Hz. Splitting patterns are indicated as follows; s, singlet; d, doublet; t, triplet; m, multiplet. The UV‐Vis absorption spectra were performed by using Agilent Cary‐3500 UV‐Vis spectrophotometer. The fluorescence emission spectra were performed by using Agilent Cary Ecsipse Fluorescence Spectrophotometer. The in vitro cell viability experimental data was measured by SpectraMax i3x. The fluorescence images were performed by using Confocal High Content Imaging and Analysis System.

### 3.3 Synthesis of endoperoxides

*Synthesis of* ***1***: Under a nitrogen atmosphere, pyridin‐2(*1H*)‐one (340 mg, 3.4 mmol) was dissolved in 10 mL of anhydrous DMF. The solution was cooled in an ice bath, and sodium hydride (60% dispersion in mineral oil, 140 mg, 3.4 mmol) was added portion wise slowly with stirring. The mixture was kept in the ice bath and stirred for an additional 15 min. Subsequently, 1‐(bromomethyl)‐4‐iodobenzene (1 g, 3.4 mmol) was added. The ice bath was then removed, and the reaction mixture was allowed to warm to room temperature and stirred for 4 h. After completion, the mixture was slowly poured into 50 mL of ice water. The resulting solution was extracted with ethyl acetate. The combined organic layers were washed with saturated brine and dried over anhydrous sodium sulfate. The solvent was removed under reduced pressure, and the crude product was purified by silica gel column chromatography. The target fractions were collected and concentrated to afford compound **1** as a yellow solid (740 mg, yield 70%). ^1^H NMR (400 MHz, Chloroform‐*d*) δ 7.55 (m, 2H), 7.20 (m, 2H), 6.95 (m, 2H), 6.54–6.45 (m, 1H), 6.05 (m, 1H), 4.96 (s, 2H). ^13^C NMR (101 MHz, CDCl_3_) δ 162.20, 139.46, 137.60, 137.14, 135.95, 129.73, 120.91, 106.18, 93.44, 51.34.

*Synthesis of* ***2***: Tris(hydroxymethyl)aminomethane (Tris, 1.21 g, 10 mmol) was dissolved in 20 mL of anhydrous methanol, and the solution was cooled to 0–5°C using an ice‐water bath. With stirring, a solution of di‐*tert*‐butyl dicarbonate (Boc_2_O, 2.4 g, 11 mmol) in methanol (10 mL) was added dropwise. After the addition was complete, the ice bath was removed, and the reaction mixture was allowed to warm to room temperature and stirred overnight. Upon completion, the mixture was concentrated under reduced pressure, yielding a crude white solid. Without further purification, the crude product was dried and weighed to afford tris((tert‐butoxycarbonyl) aminomethane) as a white solid (1.99 g, 90% yield). ^1^H NMR (400 MHz, DMSO—*d*_*6*_) δ 5.75 (s, 1H), 4.49 (t, *J* = 5.8 Hz, 3H), 3.51 (d, *J* = 5.7 Hz, 6H), 1.37 (s, 9H).

*Synthesis of* ***3***: Under an argon atmosphere, compound **2** (663 mg, 3 mmol) was dissolved in anhydrous DMF and cooled to 0°C using an ice‐water bath. With stirring, 3‐bromopropyne (2.12 g, 18 mmol) was added dropwise to the solution. The mixture was maintained at low temperature and stirred for an additional 10 min to ensure thorough mixing. Finely ground potassium hydroxide (1 g, 18 mmol) was then divided into five equal portions and added portion wise to the reaction mixture. After the addition, the ice bath was removed, and the reaction was allowed to warm to 35°C and stirred for 12 h. Upon completion, the mixture was diluted with ethyl acetate and washed with water and saturated brine. The combined organic layers were dried over anhydrous sodium sulfate. The solvent was removed under reduced pressure, and the crude product was purified by silica gel column chromatography. The target fractions were collected and concentrated to afford compound **3** as a yellow oily liquid (904 mg, 90% yield). ^1^H NMR (400 MHz, Chloroform‐*d*) δ 4.87 (s, 1H), 4.10 (d, *J* = 2.4 Hz, 6H), 3.73 (s, 6H), 2.40 (t, *J* = 2.4 Hz, 3H), 1.37 (s, 9H). ^13^C NMR (101 MHz, CDCl_3_) δ 154.61, 79.55, 79.17, 74.70, 68.78, 58.53, 57.98, 28.28.

*Synthesis of* ***4***: Under a nitrogen atmosphere, copper(I) iodide (CuI, 9.5 mg, 0.05 mmol, 10 mol %) and compound **3** (167 mg, 0.5 mmol) were dissolved in 5 mL of anhydrous triethylamine. The mixture was stirred at room temperature for 5 min. Then, palladium acetate (11.22 mg, 0.05 mmol, 10 mol%) and compound **1** (513 mg, 1.65 mmol) were added. After thorough mixed, the reaction mixture was heated to 70°C and stirred for 10 h. Upon completion, the mixture was cooled to room temperature and diluted with dichloromethane. The resulting solution was washed with 1M HCl and saturated brine. The organic layer was dried over anhydrous sodium sulfate, filtered, and concentrated under reduced pressure. The crude product was purified by silica gel column chromatography. The target fractions were collected and concentrated to afford compound **4** as a yellow oily liquid (133 mg, 30% yield). ^1^H NMR (400 MHz, Chloroform‐*d*) δ 7.38 (d, *J* = 8.4 Hz, 6H), 7.32 (m, 3H), 7.27 (dd, *J* = 6.8, 2.1 Hz, 3H), 7.19 (d, *J* = 8.5 Hz, 6H), 6.60 (d, *J* = 9.1 Hz, 3H), 6.15 (td, *J* = 6.7, 1.5 Hz, 3H), 5.10 (s, 6H), 5.04 (s, 1H), 4.36 (s, 6H), 3.89 (s, 6H), 1.39 (s, 9H).

*Synthesis of* ***5***: Compound **4** (442 mg, 0.5 mmol) was dissolved in DCM (5.0 mL) and cooled to 0–5°C. Trifluoroacetic acid (1.0 mL) was added dropwise under stirring. After the addition, the ice bath was removed and the reaction mixture was allowed to warm to room temperature, and stirred for 2 h. Upon completion, the mixture was slowly poured into a saturated aqueous sodium bicarbonate solution (20.0 mL). The resulting mixture was extracted with DCM, and the combined organic layers were washed with saturated brine. The organic phase was dried over anhydrous sodium sulfate, filtered, and concentrated under reduced pressure. The crude product was purified by silica gel column chromatography. The target fractions were collected and concentrated to afford compound 4–5 as a yellow oily liquid (352 mg, 90% yield). ^1^H NMR (400 MHz, Chloroform‐*d*) δ 7.38 (d, *J* = 7.9 Hz, 6H), 7.34–7.29 (m, 3H), 7.25 (dd, *J* = 6.8, 2.1 Hz, 3H), 7.20 (d, *J* = 8.0 Hz, 6H), 6.60 (d, *J* = 9.2 Hz, 3H), 6.15 (td, *J* = 6.7, 1.4 Hz, 3H), 5.11 (s, 6H), 4.37 (s, 6H), 3.56 (s, 6H).

*Synthesis of* ***TPy‐TPP***: Under a nitrogen atmosphere, HATU (105 mg, 0.275 mmol) and (5‐carboxypentyl) triphenylphosphonium bromide (114 mg, 0.25 mmol) were dissolved in anhydrous DMF (5 mL) and cooled to 0–5°C. With stirring, DIPEA (65 mg, 0.5 mmol) was added dropwise, and the mixture was maintained at low temperature for 15 min. A solution of compound **5** (196 mg, 0.25 mmol) in DMF was then added dropwise to the reaction mixture. After the addition was complete, the ice bath was removed, and the reaction was allowed to warm to room temperature and stirred for 4 h. Upon completion, the reaction mixture was slowly poured into ice‐cold NaHCO_3_(aq.). The mixture was extracted with DCM, and the combined organic layers were washed with NaHCO_3_(aq.) and brine. The organic phase was dried over anhydrous sodium sulfate, filtered, and concentrated under reduced pressure. The crude product was purified by silica gel column chromatography. The fractions containing the target compound were collected and concentrated to afford **TPy‐TPP** as a pale yellow solid (185 mg, 60% yield). ^1^H NMR (400 MHz, Chloroform‐*d*) δ 7.80–7.74 (m, 3H), 7.68–7.55 (m, 12H), 7.36–7.27 (m, 12H), 7.16 (d, *J* = 8.2 Hz, 6H), 6.54 (dt, *J* = 8.5, 1.5 Hz, 3H), 6.15 (td, *J* = 6.7, 1.4 Hz, 3H), 5.87 (s, 1H), 5.07 (s, 6H), 4.31 (s, 6H), 3.90 (s, 6H), 3.13–3.04 (m, 2H), 2.05 (m, 2H), 1.61–1.47 (m, 6H). ^13^C NMR (101 MHz, CDCl_3_) δ 173.05, 162.76, 139.95, 137.71, 136.94, 135.41, 133.44, 133.35, 132.24, 130.78, 130.65, 128.05, 122.30, 121.11, 118.36, 117.51, 106.75, 86.00, 85.83, 68.70, 59.63, 59.52, 51.86, 36.52, 29.61, 29.38, 24.71, 22.34, 21.83.

*Synthesis of* ***ETPy‐TPP***: In an oxygen atmosphere of 0°C, **TPy‐TPP** (122 mg, 0.1 mmol) was dissolved in 2.0 mL of DCM, and a small amount (0.1 mg) of methylene blue was added. During the reaction, red light irradiation (18 W, 625 nm LED) was used. When the reaction was completed, methylene blue was removed by adsorption with activated charcoal. After filtration, the solvent was evaporated under reduced pressure to afford ETPy‐TPP as a pale yellow solid (125 mg, 95% yield). ^1^H NMR (400 MHz, Chloroform‐*d*) δ 7.82–7.77 (m, 3H), 7.67 (m, 6H), 7.60 (m, 6H), 7.38 (d, *J* = 8.2 Hz, 6H), 7.14 (d, *J* = 8.0 Hz, 6H), 6.74 (m, 6H), 5.89 (s, 1H), 5.62–5.57 (m, 3H), 5.07–5.02 (m, 3H), 4.67 (d, *J* = 15.6 Hz, 3H), 4.49 (d, *J* = 15.6 Hz, 3H), 4.35 (s, 6H), 3.93 (s, 6H), 3.14–3.04 (m, 2H), 2.07 (t, *J* = 6.9 Hz, 1H), 1.59–1.48 (m, 6H).

### 3.4 Detection of singlet oxygen release

To detect the singlet oxygen release of endoperoxides, 1,3‐Diphenylisobenzofuran was used as a singlet oxygen probe. In a 4 mL cuvette, DMF was used as the test solvent, then DPBF (50 μM, final concentration) and **ETPy‐TPP** (250 μM, final concentration) were added and stirred; the absorbance was monitored over time using a UV‐Vis absorbance meter. DPBF alone, DPBF with **ETPy‐TPP**, and DPBF with **ETPy‐TPP** were tested. The value of *k* for the DPBF consuming rate was acquired from the absorption change at 415 nm, and the first‐order reaction kinetic formula is:

$$\ln\left[A\right]=‐\text{kt}+{\ln\left[A\right]}_{0}$$

### 3.5 MTT assay

A549, MCF‐7, HepG2, HeLa, 4T1 cells are seeded into 96‐well plates at a density of 6000 cells per well. Subsequently, cells were cultured in a carbon dioxide incubator containing 21% oxygen for 24 h. The cells were then treated with different concentrations of **TPy‐TPP** or **ETPy‐TPP** endoperoxide for 24 h. It was then incubated with 0.5 mg/mL MTT for 4 h, and finally the medium was removed and 150 μl of DMSO was added to dissolve the formazan crystals. Measure the absorbance at 570 nm with a microplate reader.

### 3.6 Detection of singlet oxygen for intracellular generation

In order to assess the intracellular singlet oxygen generation of endoperoxides, we performed fluorescence imaging by using 2′,7′‐dichlorofluorescein diacetate (DCFH‐DA) as ROS probe. A549 cells (96‐well plates at a density of 5000 cells per well) were cultured in full growth media at 37°C with 5% CO_2_ for 24 h in order to achieve proper adhesion of the cells to the glass bottomed dishes. Then incubated with different treatment: Group 1, incubated with DCFH‐DA (10 μM), Hoechst 33342 at 37°C for 30 min without any treatment (control), group 2, incubated with 30 μM **TPy‐TPP** at 37°C for 4 h, and then incubated with DCFH‐DA (10 μM), Hoechst 33342 for 30 min; group 3, incubated with 30 μM E**TPy‐TPP** at 37°C for 4 h, and then incubated with DCFH‐DA (10 μM), Hoechst 33342 for 30 min. After incubation, the media were removed and washed with PBS and imaging was observed using the High Content Imaging System.

### 3.7 Detection of mitochondrial membrane potential

To evaluate the changes in mitochondrial membrane potential, we detected the fluorescence signal changes in A549 cells stained with the JC‐1 dye. A549 cells were seeded into 96‐well plates at a density of 5000 cells per well. Subsequently, the cells were cultured in a carbon dioxide incubator containing 21% oxygen for 24 h. Then, they were incubated with different treatments: Group 1, incubated with JC‐1 for 20 min without any treatment (control); Group 2 incubated with 30 μM **TPy‐TPP** for 4 h, and then incubated with JC‐1 for 20 min; group 3, incubated with 30 μM **ETPy‐TPP** for 4 h, and then incubated with JC‐1 for 20 min. After incubation, the media were removed and washed with PBS and imaging was observed using the High Content Imaging System.

### 3.8 Detection of singlet oxygen within mitochondria

To assay the changes of singlet oxygen within mitochondria, we detected the fluorescence signal changes in A549 cells stained with the Si‐DMA. A549 cells were seeded into 96‐well plates at a density of 5000 cells per well. Subsequently, the cells were cultured in a carbon dioxide incubator containing 21% oxygen for 24 h. Then, they were incubated with different treatments: Group 1, incubated with Si‐DMA for 40 min without any treatment (control); Group 2 incubated with 30 μM **TPy‐TPP** for 4 h, and then incubated with Si‐DMA for 40 min; group 3, incubated with 30 μM **ETPy‐TPP** for 4 h, and then incubated with Si‐DMA for 40 min. After incubation, the media were removed and washed with PBS and imaging was observed using the High Content Imaging System.

### 3.9 Cell apoptosis experiment

To assay the cell apoptosis after endoperoxides treatment, we detected the fluorescence signal changes in A549 cells stained with the Annexin V‐FITC. A549 cells are seeded into 6‐well plates at a density of 8 × 10^5^ cells per well. Subsequently, the cells were cultured in a carbon dioxide incubator containing 21% oxygen for 24 h. Then incubated with different treatment for 4 h, then A549 cells were digested into a single‐cell suspension and resuspended in Annexin V binding buffer. Then Annexin V‐FITC and PI were added to stain for 15 min. The data was obtained by flow cytometer.

### 3.10 Live/dead cell staining using Calcein‐AM/PI

The live/dead staining assay was performed to visualize the status of A549 cells after being treated by different samples. A549 cells are seeded into 96‐well plates at a density of 6000 cells per well. Subsequently, the cells were cultured in a carbon dioxide incubator containing 21% oxygen for 24 h. Then incubated with different treatment for 4 h, washed by PBS. Then Calcein‐AM/propidium iodide staining reagents were added and incubated for 20 min. The cells were observed by High Content Imaging System.

### 3.11 3D (three‐dimensional) tumor spheres

A549 cells were used to generate tumor spheroids. Single‐cell suspensions (with desired seeding density) were seeded into Nunclon™ Sphera™ 96‐well‐low attachment plates (Thermo Fisher Scientific) in DMEM‐high glucose supplemented with 10% FBS and 1% penicillin/streptomycin. The low attachment plates were incubated for 3 days (37°C, 5% CO2) and incubated with different treatments for 4 h. Then, tumor spheroids were washed by PBS, and Calcein‐AM/PI was added to stain for 30 min. After incubation, the media were removed and washed with PBS and imaging was observed using the High Content Imaging System.

### 3.12 Colony formation assays

A549 cells were seeded at a density of 1000 cells/well in a six‐well plate. The next day, the A549 cells were observed to be fully adherent to the cell wall and were incubated with different treatments. The fluid was changed every 3 days, the cell mass (counting ≥50 cells) was observed under the microscope, the cell mass was visible to the naked eye on the cell culture dish, and cell cloning was performed. Cells in each well were fixed with 4% paraformaldehyde for 20 min, washed three times with PBS, stained with 0.1% crystal violet solution for 15 min, washed again with PBS three times, dried, and photographed.

### 3.13 Wound healing scratch assay

A549 cells were seeded in 6‐well plates (8 × 10^5^ cells/well) and after 24 h with acceptable confluency indicating a monolayer (∼80–90% confluency), 3 parallel scratches were made on the bottom of the dish using a 10 μL pipette tip. Thereafter, the detached cells were removed with PBS and immediately the cells were treated with the effective concentrations of **TPy‐TPP**/**ETPy‐TPP** (30 μM) and/or control (medium containing the equal amount of test compounds solvent). Scratch photographs were taken at marked positions after 0 h, 24 h, and 48 h using an inverted fluorescence microscope. The scratch distances were quantified using ImageJ.

### 3.14 Transwell migration assay

A549 cells were resuspended in serum‐free medium and seeded into the upper chambers of a 24‐well Transwell plate at 1 × 10^5^ cells per well in 150 μL. The lower chambers received 600 μL of DMEM containing 10% FBS as a chemoattractant. After careful assembly to avoid bubbles, the plate was incubated at 37°C with 5% CO_2_ for 24 h. The upper chamber medium was then replaced with serum‐free medium containing 30 μM ETPy‐TPP or TPy‐TPP for the experimental groups, while the controls were untreated. After another 24 h, non‐migrated cells were removed from the upper membrane surface with a cotton swab. Migrated cells on the lower surface were fixed with 4% PFA, stained with 0.1% crystal violet, and imaged under an inverted microscope for quantification with ImageJ software.

## 4 CONCLUSION

In this work, the results that we obtained clearly showed that targeting mitochondria with 2‐pyridone endoperoxides and multivalent sources of singlet oxygen offer great advantage in anti‐cancer activity in 2D and 3D cell cultures and as revealed by both MTT and colony formation assays. Fluorescence imaging strongly corroborates our understanding of efficient singlet oxygen delivery by the endoperoxide. We are confident that multivalent sources of singlet oxygen will find practical utility in the therapeutic protocols in treating cancer. Our work along these lines is in progress.

## CONFLICT OF INTEREST STATEMENT

The authors declare no conflicts of interest.

## ETHICS STATEMENT

No animal or human experiments were involved in this study.

## Supporting information

Figures S1–S10

## Data Availability

The data that supports the findings of this study are available in the supplementary material of this article.
