# Supplementary material for: Targeting cancer cell mitochondria with a multivalent source of singlet oxygen
Source: Smart Mol. 2026 Feb 10;4(1):e70035. doi: 10.1002/smo2.70035 (PMC13104100; doi:10.1002/smo2.70035)
Supplement: Supplementary file 1 — Figures S1–S10 [file SMO2-4-e70035-s001.docx]

Supporting Information

Targeting Cancer Cell Mitochondria with a Multivalent Source of Singlet Oxygen

Wanwan Wang,^1^ Lei Wang,^1^* Rensong Sun,^1^ Shoucai Yan^1^ and Engin U. Akkaya^1,2^*

**Fig S1.** Temporal evolution of **ETPy-TPP** ^1^H NMR in CDCl_3_ at 37 ℃. (Spectrum 1 - 0 hours, Spectrum 2 - 1 hours, Spectrum 3 - 2 hours, Spectrum 4 - 4 hours, Spectrum 5 - 6 hours, Spectrum 6 - 8 hours, Spectrum 7 - 10 hours.

**Fig S2.** Half-life calculation of DPBF with **ETPy-TPP**: 4.5 hours (at 37 ℃ in CDCl_3_)

**Fig S3.** Time dependent UV-Vis spectra of DPBF in the presence of **ETPy-TPP** in DMF at 37 ℃.

**Fig S4.** Calculation of the DPBF consumption rate: *k* = 2 × 10^−4^ s^−1^

**Fig S5.** Time dependent UV-Vis spectra of DPBF in the presence of **TPy-TPP** in DMF at 37 ℃.

**Fig S6.** Time dependent UV-Vis spectra of DPBF in DMF at 37 ℃.


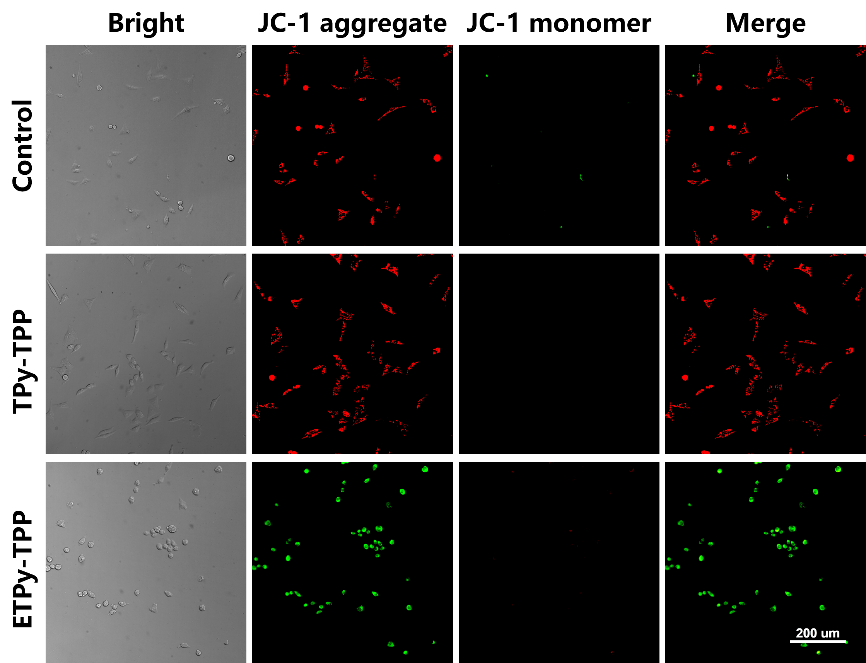


**Fig S7.** JC-1 used as a probe for detecting changes in mitochondrial membrane potential. First row : Control, second row : incubation with 30 µM **TPy-TPP**, third row : incubation with 30 µM **ETPy-TPP**.


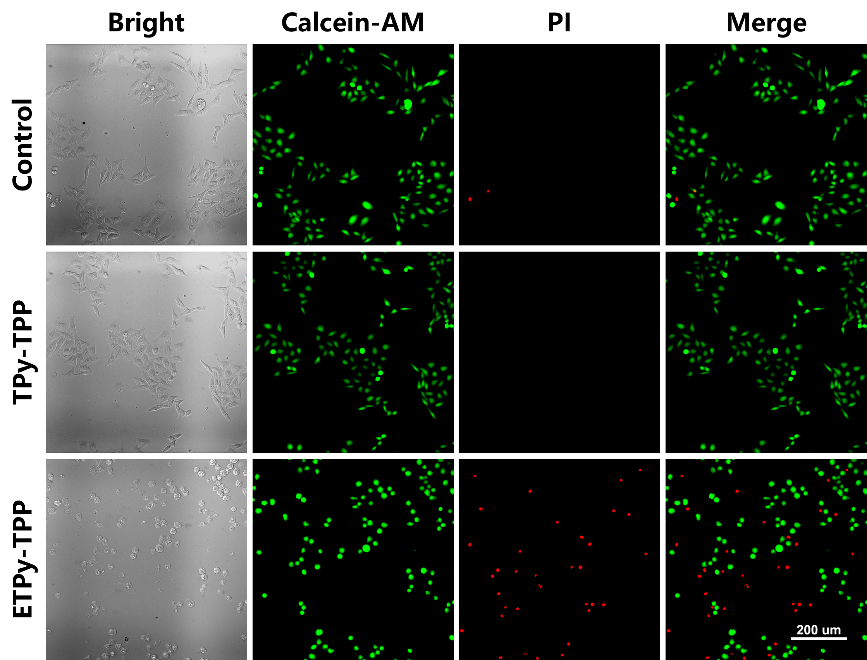


**Fig S8.** Dead/live staining images with different treatments. First row: Control, second row: incubation with 30 µM **TPy-TPP**, third column : incubation with 30 µM **ETPy-TPP**.


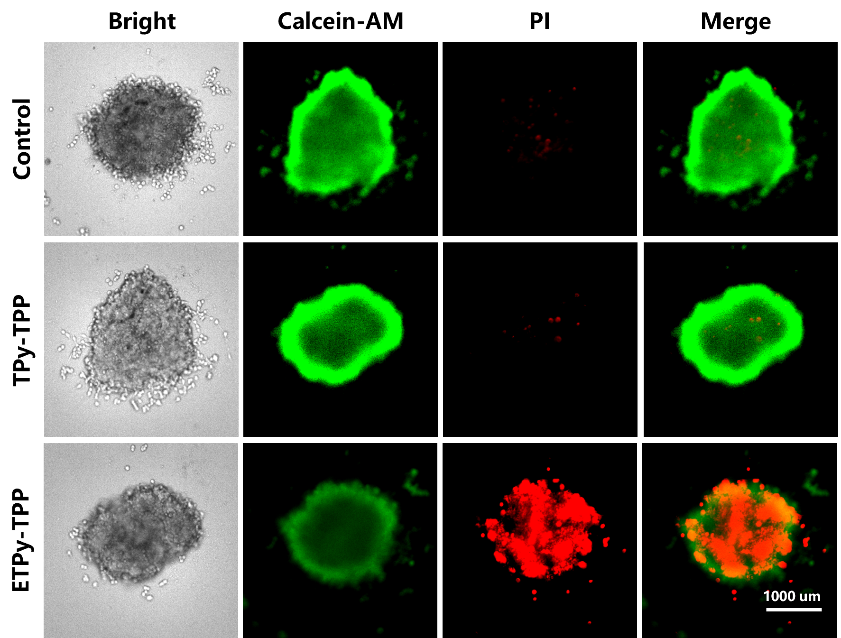


**Fig S9.** Dead/live staining images with different treatments A549 cell-derived tumor spheroids. First row: Control, second row: incubation with 30 µM **TPy-TPP**, third column : incubation with 30 µM **ETPy-TPP**.


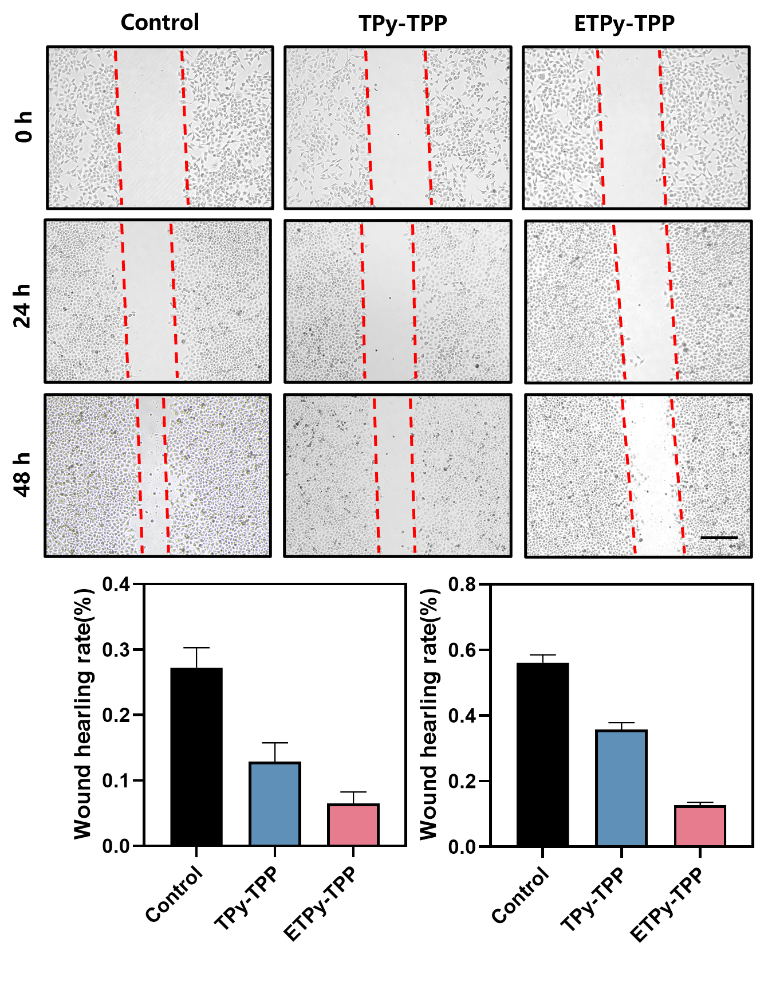


**Fig S10.** Micrographs of cell migration and wound healing rate were scratched and treated with endoperoxide **ETPy-TPP** and compound **TPy-TPP**. Scale, 100 μm.

Fig. S11 NMR Spectra

^1^H NMR of compound **1**

^13^C NMR of compound **1**

^1^H NMR of compound **2**

^1^H NMR of compound **3**

^13^C NMR of compound **3**

^1^H NMR of compound **4**

^1^H NMR of compound **5**

^1^H NMR of compound **TPy-TPP**

^13^C NMR of compound **TPy-TPP**

^1^H NMR of compound **ETPy-TPP**
